# Fighting biofilm: bacteriophages eliminate biofilm formed by multidrug-resistant *Enterobacter hormaechei* on urological catheters

**DOI:** 10.1007/s00430-025-00844-0

**Published:** 2025-07-03

**Authors:** Martyna Cieślik, Michał Wójcicki, Paweł Migdał, Ilona Grygiel, Olaf Bajrak, Filip Orwat, Andrzej Górski, Ewa Jończyk-Matysiak

**Affiliations:** 1 Bacteriophage Laboratory, Hirszfeld Institute of Immunology and Experimental Therapy, Polish Academy of Sciences, Weigla 12, 53-114 Wroclaw, Poland; 2 Inter-Departmental Laboratory of Instrumental Analysis and Preparation, Hirszfeld Institute of Immunology and Experimental Therapy, Polish Academy of Sciences, Weigla 12, 53-114 Wroclaw, Poland; 3 Phage Therapy Unit, Hirszfeld Institute of Immunology and Experimental Therapy, Polish Academy of Sciences, Weigla 12, 53-114 Wroclaw, Poland; 4 Professor Emeritus, Department of Immunology, Medical University of Warsaw, Nowogrodzka 59, 02-006 Warsaw, Poland

**Keywords:** Lytic bacteriophages, Bacterial biofilm, Urological catheters, *Enterobacter cloacae* complex, ESKAPE pathogens, Metal nanoparticles

## Abstract

The *Enterobacter cloacae* complex (ECC) is a prevalent nosocomial pathogen associated with various human infections, which currently comprises several species, including *Enterobacter cloacae* and *Enterobacter hormaechei*. Strains capable of producing biofilm on various biotic and abiotic surfaces pose a particular threat. Therefore, we focused on three *E. hormaechei* strains in whose genomes the presence of the biofilm-related genes: *fimA*, *csgA*, *csgD*, and *sdiA* was confirmed. Kinetic of biofilm formation by these strains on urological catheters depended on the catheter material (silicon or latex), temperature (24 °C or 37 °C) and incubation time. The ability of phages to disrupt biofilm formation was assessed and found to be the most effective when phages were applied at an early stages of this process. Moreover, destruction of existing biofilm by bacteriophages and/or silver or copper nanoparticles was strain-dependent. Incubation with *Enterobacter*-specific bacteriophages enabled, in some cases, almost complete eradication of three-day biofilms attached to urological catheters. In genomes of two *Enterobacter*-specific bacteriophages the presence of regions encoding proteins with lytic activity were identified (6 regions in Entb_43 phage and 4 regions in Entb_45 phage genomes, respectively). These results highlight the threat of biofilm-related infections, but also indicate the multifaceted anti-biofilm activity of bacteriophages, which should be considered for useful in clinical practice.

**Supplementary Information:**

The online version contains supplementary material available at 10.1007/s00430-025-00844-0.

## Introduction

According to the updated World Health Organization (WHO) Bacterial Priority Pathogens List (2024), third-generation cephalosporin-resistant *Enterobacterales* and carbapenem-resistant *Enterobacterales* belong to the critical group, which is characterized by high mortality and morbidity, high transmissivity, and limited treatment options [1]. *Enterobacter* spp. are Gram-negative rods belonging to the *Enterobacteriaceae* family and *Enterobacterales* order, responsible for various infections [2]. Furthermore, *Enterobacter* spp. is also a member of ESKAPE pathogens (*Enterococcus faecium*, *Staphylococcus aureus*, *Klebsiella pneumoniae*, *Acinetobacter baumannii*, *Pseudomonas aeruginosa*, *Enterobacter* spp.)—group of bacteria primarily identified as a critical multidrug-resistant (MDR) and represent a global threat to health [3–5]. Healthcare-associated infections (HAIs) caused by *Enterobacter* include urinary tract infections (UTIs), surgical wounds, respiratory tract, osteoarticular system, central nervous system, and cardiovascular system infections, as well as bacteremia and sepsis [6, 7]. Moreover, these bacteria are also component of the natural microbiota of animal and human gastrointestinal tract [8]. Additionally, food contamination with pathogenic *Enterobacter* strains are also common [9, 10].

The *Enterobacter cloacae* complex (ECC) is a prevalent nosocomial pathogen associated with various human infections, such as bacteremia, as well as infections of the respiratory tract, wounds, and urinary tract. The ECC currently comprises several species, including *Enterobacter cloacae*, *Enterobacter hormaechei*, *Enterobacter kobei*, *Enterobacter asburiae*, *Enterobacter cancerogenus*, *Enterobacter nimipressuralis*, and *Enterobacter mori* [11, 12]. Recently identified species, such as *Enterobacter roggenkampii*, *Enterobacter chengduensis*, and *Enterobacter bugandensis*, have also been grouped within the ECC [13]. Among these, *E. cloacae* and *E. hormaechei* are the most commonly isolated species from human clinical samples [11, 12].

It is believed that the first observations of bacterial biofilm were made already by Anthony van Leeuwenhoek at the turn of the 17th and 18th centuries [14]. Microbial biofilms form an advanced multicellular anatomical structure that determines many of their functions [15]. The biofilm formation process occurs in multiple stages, starting with the reversible and then irreversible attachment of planktonic bacteria to the surface, through the formation of microcolony and its maturation together with dead, persistent, and sessile bacterial cells, to its dispersion [16]. Biofilm formation and existence also acquires the presence of a self-produced extracellular polymeric substance (EPS) matrix, containing polysaccharides, extracellular DNA and proteins (including enzymes), lipids, as well as water which is the largest component of EPS [16, 17]. Infections caused by *Enterobacter* spp. (the most common of which is *E. cloacae*) are also associated with biofilm formation on various surfaces, including abiotic ones such as medical devices, however, most studies focus on other biofilm-forming members of the *Enterobacteriaceae* family [18, 19]. Special attention is paid to catheter-related infections associated with the formation of biofilms, which are not only difficult to detect but also difficult to treat and prevent [17, 20]. Catheter-associated UTIs constitute a large part of nosocomial infections [21, 22].

MDR strains of biofilm-forming bacteria are particularly dangerous for immunocompromised patients, e.g., transplant recipients undergoing immunosuppressive treatment [23]. Strong biofilm producers are also known to influence the pro-inflammatory activation of macrophages [24]. Furthermore, cell-free biofilm components have been shown to induce a pro-inflammatory effect, for example, by increasing the secretion of IL-6 by human keratinocytes [25]. Importantly, biofilms tend to be more resistant to antibiotics than planktonic bacteria [26, 27]. This is a key factor contributing to the failure of conventional clinical treatments for infectious diseases. At present, there are no drugs specifically targeting bacterial biofilms in clinical practice, highlighting the urgent need for the development of new preventive and therapeutic strategies to effectively treat biofilm-associated bacterial infections [17].

Bacteriophage therapy, introduced in the early 20th century [28], involves the use of phages—viruses specific to bacteria—to eliminate harmful bacterial pathogens, including those associated with infectious diseases [29, 30]. Bacteriophages have the ability to lyse bacteria, leading to bacterial cell death rather than merely limiting their growth. When infecting bacteria, phages are capable of self-amplification, enabling effective infection control without the need for high doses [31]. Composed primarily of proteins and nucleic acids, phages are non-toxic to eukaryotic cells [32]. Although phages may elicit immune reactions, purified phage preparations are recommended to be used to minimize the risk of anaphylactic reactions [33]. Due to their host specificity, phages—unlike most broad-spectrum antibiotics—have only minimal impact on the physiological bacterial microbiota [32, 34].

Bacteriophage-based approaches are increasingly recognized as a viable means of targeting bacterial biofilms, particularly those produced by MDR pathogens that are difficult to eradicate using conventional antibiotics [35]. Having co-evolved with bacterial biofilms, bacteriophages employ at least four mechanisms to disrupt them: (a) they can compromise the biofilm's integrity by targeting and lysing bacterial cells responsible for producing the biofilm matrix; (b) while diffusing through the biofilm, bacteriophages can carry or produce depolymerizing enzymes that break down the EPS; (c) phages may induce the expression of depolymerizing enzymes encoded in the bacterial host genome to degrade EPS; and (d) in resistant bacterial cells, phages can remain in dormant state (lysogeny or pseudolysogeny) until conditions become favourable, ultimately leading to bacterial host cell lysis [36]. Another approach to biologically disrupting biofilms is the use of purified phage enzymes. Phage genomes, particularly those of lytic phages, encode numerous genes for enzymes specifically adopted to degrade the biofilm matrix. These enzymes typically target the bacterial cell wall during phage release from the host and possess the ability to break down the exopolysaccharides that make up the biofilm structure [36, 37].

In recent years, searching for and characterization of bacteriophages against MDR *E. cloacae* complex strains attract more attention [38–43]. Therefore, the aim of this study was to evaluate the effectiveness of single *Enterobacter*-specific phages and their cocktail in eradication of biofilms formed by clinical MDR *E. hormaechei* strains belonging to the ECC.

## Materials and methods

### The biofilm-forming ability of bacterial strains

Biofilm-forming ability was investigated on twenty MDR *Enterobacter* strains (*E. cloacae*, *E. hormaechei*, and *E. kobei*) isolated from patients who submitted to experimental bacteriophage therapy at the Hirszfeld Institute of Immunology and Experimental Therapy, Polish Academy of Sciences (HIIET PAS), and collected during years 2017–2021, and were used in our previous study [44]. The bacterial strain species were identified using matrix-assisted laser desorption/ionization time-of-flight mass spectrometry (MALDI-TOF MS) (Bruker Daltonics, Billerica, MA, USA).

To assess biofilm-forming ability, 200 µL of a 3–4-hour bacterial suspension in sugar broth was diluted 500× (to reach a titer of approximately 10^5^ colony forming units per milliliter (CFU/mL)), placed in flat-bottomed 96-well plates in 8 replicates and incubated at 37 °C. The negative control was the same volume of bacterial culture medium. After 24 or 48 h, solutions were gently removed, wells were washed twice with phosphate-buffered saline (PBS) and dried at 60 °C for 40 min or at 37 °C for 90 min. Next, 190 µL of 1% crystal violet (Chempur, Piekary Śląskie, Poland) solution (w/v) was applied for each well, incubated at room temperature in the dark for 20 min, washed twice with miliQ water, dried at room temperature, and then 200 µL of 95% ethanol was added and incubated on the laboratory counter. After 30 min, 100 µL of each solution was transferred to a new plate and absorbance was measured spectrophotometrically (λ = 570 nm) using a microplate reader (Sunrise; Tecan, Switzerland) [45–48]. The ability to produce biofilm was assessed according to Stepanović et al. [49].

Three bacterial strains that produced biofilm in varying intensity and were sensitive to at least one phage (*E. hormaechei* strain 30345—bacterial host for Entb_43 phage, sensitive to both phages, was isolated from fistula; *E. hormaechei* strain 29796—bacterial host for Entb_45 phage, sensitive to both phages, isolated from urine; and *E. hormaechei* strain 30528, sensitive only to Entb_45 phage, was isolated from pus) were selected for further studies.

### Bacterial strains and detection of biofilm-related genes

Bacterial DNA was isolated using a commercial DNeasy PowerFood Microbial Kit (Qiagen, GmbH, Hilden, Germany), according to the manufacturer’s protocol. The taxonomic identification of the studied strains as *E. hormaechei* was confirmed through the amplification of the conserved region of the *16S* rDNA gene. For each strain, the presence of main markers (i.e., *fimA*, *fimH*, *fliC*, *csgA*, *csgD*, and *sdiA* genes) associated with biofilm formation was determined. The primer sequences, gene products studied, and PCR (Mastercycler Nexus GX2 thermocycler; Eppendorf, Hamburg, Germany) reaction parameters are presented in Table 1. The obtained amplicons were separated by electrophoresis on a 2% agarose gel containing the 1% of ethidium bromide solution (2 μL/100 mL; Merck, Darmstadt, Germany). To determine the size of the amplicons, 5 μL of a DNA ladder ranging from 100 to 10,000 bp was used (Thermo Fisher Scientific, Vilnius, Lithuania). Electrophoresis was performed at 80 V for 60 min using the Horizontal Electrophoresis System (Hoefer PS 300-B; San Francisco, CA, USA). The bands were visualized with the Azure 200 Bioanalytical Imaging System (Azure Biosystems Inc.; Dublin, CA, USA). Sanger sequencing of the *16S* rDNA gene was performed by Genomed SA (Warsaw, Poland). The raw sequences were analyzed using BLASTn (National Center for Biotechnology Information, NCBI), submitted, and deposited in the GenBank database.

**Table 1:** PCR reaction conditions for the amplification of genes that are markers of biofilm formation and the conserved *16S* rDNA region for taxonomy identification of *Enterobacter* strains

Target gene	Gene product (function)	Primer sequences 5’–3’	Product size	PCR conditions	References
*16S* rDNA	Conserved region in bacteria (enables bacteria identification)	F: AGAGTTTGATCCTGGCTCAG R: ACGGCTACCTTGTTACGACT	1458 bp	Initial denaturation step of 2 min at 95 °C, followed by 35 cycles as follows: 30 s at 94 °C for denaturation, 35 s at 51 °C for annealing, 1 min at 72 °C for extension, and a final extension step of 10 min at 72 °C	[50]
*fimA*	Major subunit of type 1 pili (cell adhesion and biofilm formation)	F: TGCTGTCGAGGATCTCAATG R: ACGGTTAATCTCGGCCAGTA	229 bp	Initial denaturation step of 5 min at 95 °C, followed by 35 cycles as follows: 30 s at 94 °C for denaturation, 30 s at 58 °C for annealing, 1 min at 72 °C for extension, and a final extension step of 10 min at 72 °C	[51]
*fimH*	Major adhesin (auto aggregation and biofilm formation)	F: TGCAGAACGGATAAGCCGTGG R: GCAGTCACCTGCCCTCCGGTA	508 bp	Initial denaturation step of 5 min at 95 °C, followed by 35 cycles as follows: 30 s at 94 °C for denaturation, 45 s at 63 °C for annealing, 1 min at 72 °C for extension, and a final extension step of 10 min at 72 °C	[52]
*fliC*	Flagella (biofilm formation, adhesion, and cell motility)	F: ACTCAGGCTTCCCGTAACGC R: GGCTAGTATTGTCCTTATCGG	763 bp	Initial denaturation step of 5 min at 95 °C, followed by 35 cycles as follows: 30 s at 94 °C for denaturation, 30 s at 55 °C for annealing, 1 min at 72 °C for extension, and a final extension step of 10 min at 72 °C	[53]
*csgA*	Curlin subunit A (cell adhesion, aggregation, and biofilm formation)	F: TTCAAAGTGGCAGTTATTGCAG R: TTTTTGCAGCAGATCGATAGAA	276 bp	Initial denaturation step of 5 min at 95 °C, followed by 30 cycles as follows: 30 s at 94 °C for denaturation, 30 s at 56 °C for annealing, 30 s at 72 °C for extension, and a final extension step of 10 min at 72 °C	[54]
*csgD*	Curlin subunit D (curli assembly, transport, and structural components, important for biofilm formation)	F:GAAATTGCATAATATTCAACGTTC R: TTTGTTCAGGATCTCTTTTTCAC	385 bp	Initial denaturation step of 5 min at 95 °C, followed by 30 cycles as follows: 30 s at 94 °C for denaturation, 30 s at 54 °C for annealing, 30 s at 72 °C for extension, and a final extension step of 10 min at 72 °C	[54]
*sdiA*	LuxR-type, quorum sensing (QS) transcription regulator (pili production, biofilm formation, adhesion, and cell motility)	F: TTAGCGTTCAATTTGCTCCAGATG R: GATATCAGTCAGATAAGCCCCGTC	951 bp	Initial denaturation step of 5 min at 95 °C, followed by 30 cycles as follows: 30 s at 94 °C for denaturation, 30 s at 55 °C for annealing, 1 min at 72 °C for extension, and a final extension step of 10 min at 72 °C	[55]

### Time- and temperature-dependent biofilm forming on urological catheters

Sterile 4 mm diameter silicon and latex Foley catheters were aseptically cut into 2 mm thick discs and introduced to 5 mL of sugar broth in a glass tubes inoculated with a specific bacterial suspension (10^5^ CFU/mL, based on plots of dependence of the bacterial titer on OD_600_) [56, 57]. According to Misra et al. [18], incubation was carried out at 24 °C (room temperature) or 37 °C for different periods (24 h, 48 h, 72 h, 96 h, or 1 week) under static conditions. At the same time intervals, the optical density of planktonic bacterial cultures was measured [56]. After a respective time of incubation, discs were washed thrice with PBS on sterile cell culture dishes, dried for 4–5 h in laminar airflow, and separately transferred into wells on 96-well plates, in 8 replicates of each catheter for one bacterial strain at a given temperature, and stained as described above. After rinsing, the discs were gently blotted with sterile gauze to remove the solution from the lumen by capillary action and then dried at 37 °C. After dissolving the biofilm in 95% ethanol, catheter discs were removed, 100 µL of each solution was transferred into a new plate, and the absorbance was measured (λ = 570 nm). Values from negative controls carried out separately for both catheters were subtracted from the values of the test samples.

### Phage amplification

Two lytic *Enterobacter*-specific bacteriophages, Entb_43 and Entb_45, were characterized biologically, morphologically, and genetically in our previous study [44]. The best phage-to-bacteria ratio (0.01 or 0.001, respectively) was used for bacteriophage amplification. Briefly, phage lysate and appropriate bacterial suspension (*E. hormaechei* strain 30345 is the primary host for Entb_43 phage; *E. hormaechei* strain 29796 is the primary host for Entb_45 phage) were added to 250 mL of peptone water. After overnight incubation at 37 °C and filtration with the use of syringe bacteriological filters (pore diameter of 0.22 µm) (Millipore, Burlington, MA, USA), phage titers were calculated using routine test dilution (RTD) and double-layer agar methods.

### Screening of phage genomes for lysis-related phage enzymes

To search for lysis-related phage enzymes, the Reference Sequence (RefSeq) v6.7 database [58] at NCBI was used.

### Prevention of biofilm formation by phages

To assess whether phages alone or in a phage cocktail added to the biofilm during its formation can inhibit or disturb this process, 200 µL of 3–4-hour bacterial suspensions (10^5^ CFU/mL) were placed on 96-well plates. During the 24-hour incubation at 37 °C, at 0 h, 2 h, 4 h, 6 h, or 8 h, 100 µL of suspension was taken from each well, followed by a single addition of 100 µL of phages (10^7^ PFU/mL), or medium in the positive controls. The selected bacteriophage titer was determined based on its demonstrated effectiveness in prior assays, such as the spot test and inhibition of planktonic bacterial growth in liquid culture [44]. At the same time, positive control samples with bacteria only and negative controls without the addition of bacteria and phages were cultured for 24 h. After incubation, all steps described above have been completed to stain biofilm and assess the ability for biofilm formation. The experiments were conducted with 14–16 replicates for each group.

### Biofilm degradation by phages and/or silver and copper nanoparticles

After 18–20 h incubation of 200 µL bacterial suspension (10^5^ CFU/mL) on 96-well plates and washing twice with PBS, the plates were dried as described. Next, 200 µL of one of the following agents was added to the wells in a single dose: Entb_43 phage (10^7^ PFU/mL), Entb_45 phage (10^7^ PFU/mL), a cocktail of both phages, silver nanoparticles, copper nanoparticles (at two concentrations: 5 or 50 mg/kg (ppm)), or a mixture of each phage with each nanoparticle, in 4 repetitions. Positive controls included the addition of bacterial suspension, and negative controls included the addition of peptone water only. After 18–20 h of incubation at 37 °C, the staining procedures described above were performed and the percentage degradation of bacterial biofilm was calculated according to the formula [59]:

$$\% \deg = \left( {1 - \frac{{OD_{treated} }}{{OD_{untreated} }}} \right)*100\%$$

### Scanning Electron Microscope (SEM) imaging

Bacterial biofilms formed on polystyrene 96-well plates, as well as biofilms incubated for 24 h with selected bacteriophages, were imaged using SEM. The following samples were selected for imaging: biofilm produced by *E. hormaechei* strain 30345 before and after exposure to Ent_43 phage; biofilm produced by *E. hormaechei* strain 29796 before and after exposure to Entb_45 phage; and biofilm produced by *E. hormaechei* strain 30528 before and after exposure to a cocktail of both phages. Biofilms were scraped from the wells, centrifuged to remove residual milliQ water after rinsing, and suspended in 1 mL of a 2.5% glutaraldehyde solution (Merck, Darmstadt, Germany). Sample preparation and imaging were performed according to previously described protocols [60] with the use of Scanning Electron Microscope Auriga 60 (Oberkochen, Germany).

### Degradation of biofilm attached to catheters by phages

The biofilms selected for the study were grown at 37 °C for 3 days (72 h) on both latex and silicone urological catheters. Subsequently, catheters were gently washed twice with PBS and introduced to phage lysates (with a titer of 10^7^ PFU/mL), phage cocktail, or fresh peptone water in positive controls. Crystal violet staining and measurement of the absorbance were performed according to the procedures described above.

### Statistical analysis

The graphical presentations of results and the statistical analysis were performed using the GraphPad Prism v9.4.1 (GraphPad Software Inc., San Diego, CA, USA) software. A one-way ANOVA with Tukey's or Dunnett's multiple comparisons test was used to determine the statistical significance of differences between groups.

## Results and discussion

### *Enterobacter* strains produce biofilms on abiotic surfaces

**Fig. 1:**
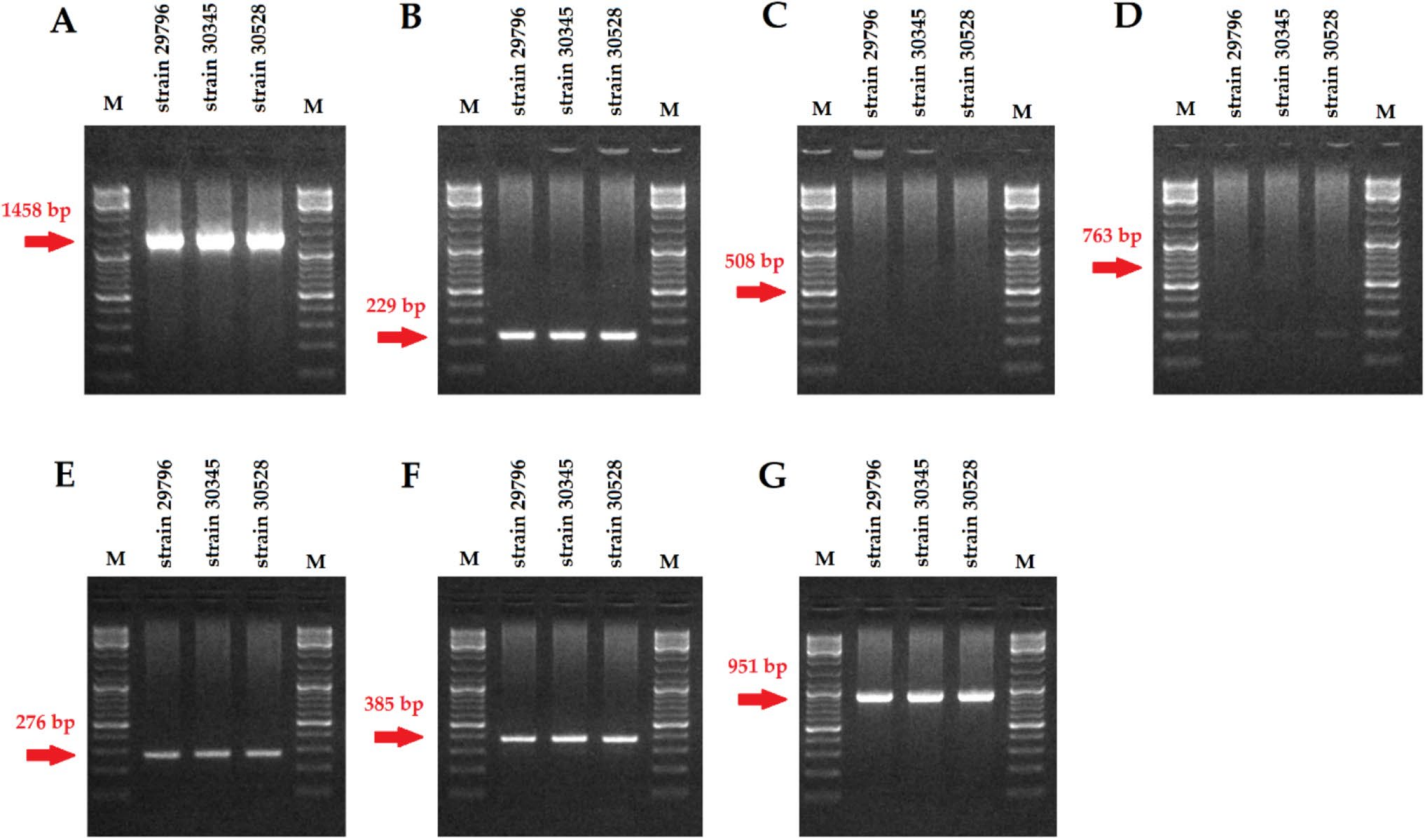
Detection of biofilm formation marker genes and the conserved *16S* rDNA region for taxonomic identification of *Enterobacter* strains: **A** *16S* rDNA, **B** *fimA*, **C** *fimH*, **D** *fliC*, **E** *csgA*, **F** *csgD*, **G** *sdiA*. The mass marker lane (in the range of 100–10,000 bp) is marked with the letter M

We assessed the ability of 20 *Enterobacter* strains to form biofilms, which were also used to characterize the bacteriophages described in previous studies [44]. Most strains were weak biofilm producers and this ability generally did not change after prolonged incubation (with some exceptions) (Supplementary Table S1). Three *E. hormaechei* strains were selected for further study: (1) *E. hormaechei* strain 30345, which produced a moderate biofilm and is primary host for Entb_43 phage; (2) *E. hormaechei* strain 29796, which produced a weak biofilm and is primary host for Entb_45 phage; (3) *E. hormaechei* strain 30528, which produced a weak biofilm and is sensitive only to one tested phage (Supplementary Table S1). These strains were initially classified as *E. cloacae* (based on MALDI-TOF MS), but analysis of the *16S* rDNA gene region (performed in this study; products separated on agarose gel are shown in Fig. 1A) suggests a closer similarity of the strains to the species *E. hormaechei*. The *16S* rDNA gene region sequences have been deposited in the GenBank database under accession numbers PQ785782, PQ785787 and PQ785788 for *E. hormaechei* strain 29796, *E. hormaechei* strain 30345 and *E. hormaechei* strain 30528, respectively. Both bacterial hosts were sensitive to both phages tested. The last strain insensitive to one of the phages was selected to assess whether both phages would be able to degrade or inhibit the biofilm produced by this strain, even though one of them did not destroy the bacteria in the spot test.

The presence of the *fimA*, *csgA*, *csgD*, and *sdiA* genes was confirmed in all three strains tested (Fig. 1B, E, F, G, respectively). Conversely, the *fimH* and *fliC* genes were not detected in any of the strains (Fig. 1C, D, respectively).

The *fimA* and *fimH* gene products—the FimA and FimH proteins—are essential for *Enterobacter* adhesion [61]. The presence of *fimA* gene, which encodes a type 1 fimbriae (pili) adhesion structure, enables bacteria to invade host tissues, form biofilms, and prevent the entry of antibiotics into the cells [62]. The major adhesin, FimH, encoded by the *fimH* gene, plays a crucial role in pathogenesis and contributes to biofilm formation on catheter surfaces [61, 63]. The *csgA* gene encodes for the major structural subunit of curli fimbriae (curlin subunit A) [61]. CsgA is a key factor in cell adhesion, aggregation, and biofilm formation [54]. The CsgD protein, also known as curlin subunit D, is a transcriptional regulator that controls various factors involved in curli assembly, secretion, and structural components, all of which are essential for biofilm formation [62]. The *fliC* gene encodes a universal flagella subunit that causes the cell motility of *Enterobacter* and thus promotes adhesion and biofilm formation [53, 64]. SdiA, a transcription factor belonging to the LuxR family, regulates gene expression in response to AHLs produced by other bacterial species, suggesting a form of interspecies cell-cell communication mediated by this factor [65]. The SdiA regulator has been implicated in pathogenesis by modulating fimbria expression, biofilm formation, and the production of QS autoinducers [66].

**Fig. 2:**
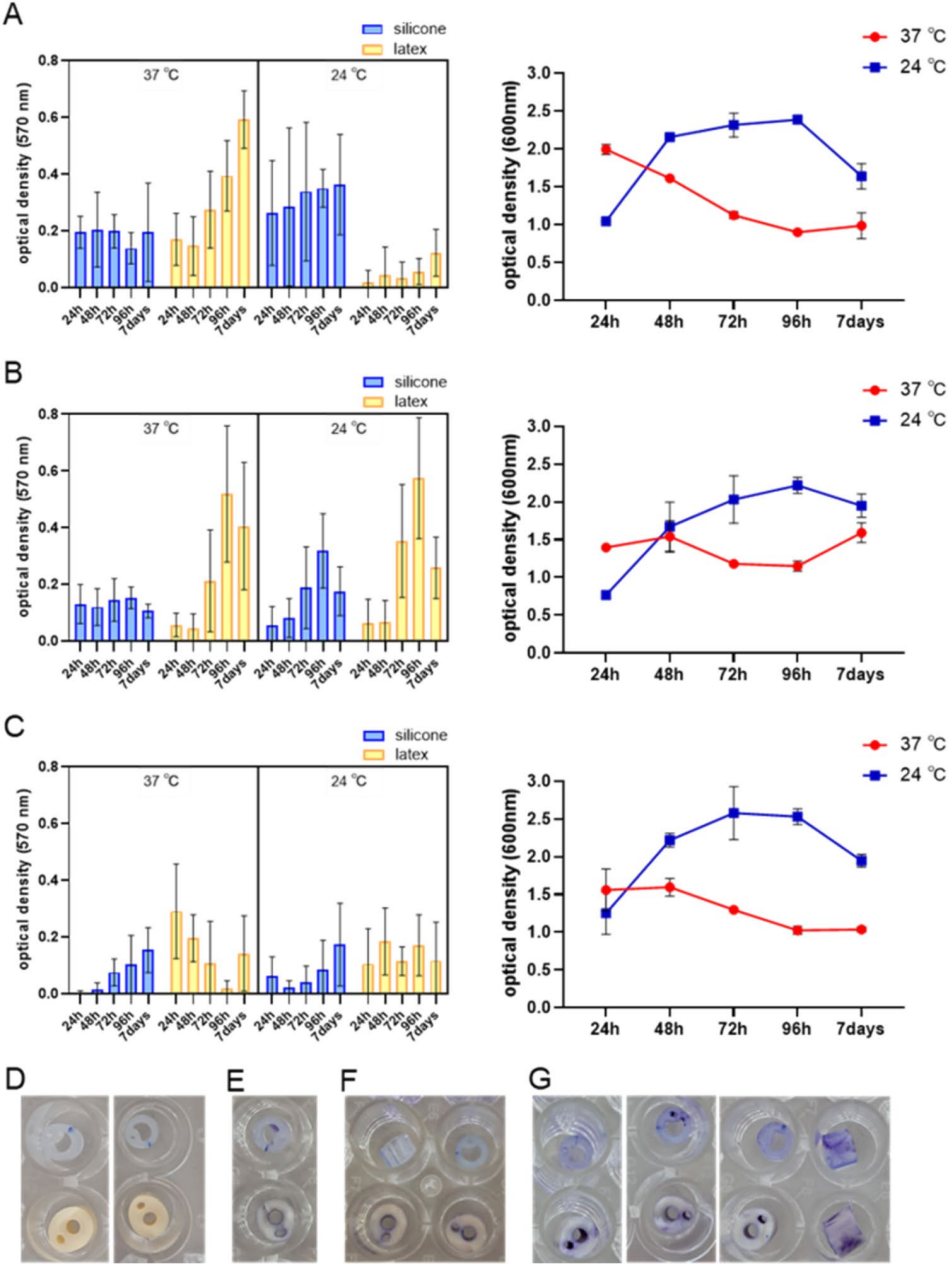
Biofilm-forming ability of three *Enterobacter* isolates: **A** *E. hormaechei* strain 30345, **B** *E. hormaechei* strain 29796, **C** *E. hormaechei* strain 30528. The left graphs show the degree of biofilm formation on silicon or latex urological catheters, depending on temperature (24 °C or 37 °C), for different incubation times (24 h, 48 h, 72 h, 96 h, or 7 days; *n* = 6 for each group). The right graphs represent kinetic growth of planktonic bacteria in the same temperatures and time periods (two biological replicates, each of them with three technical replicates). The lower panel shows the intensity of biofilm staining on silicone and latex catheters: **D** unstained, at the beginning of the experiment; **E** after 24 h, **F** after 72 h, or **G** after 7 days of incubation with bacterial suspension. The pictures show 2 mm long catheter discs with adhered biofilm after staining with crystal violet, rinsing and drying. Error bars represent the standard deviation (± SD) of the mean measured optical density

The kinetics of biofilm formation by three selected strains were evaluated on Foley urological catheters made of silicon or latex, depending on two different temperatures (24 °C, corresponding to room temperature, at which catheters usually are stored, and 37 °C, corresponding to body temperature), for 1 week. Catheter samples were collected after 24 h, 48 h, 72 h, 96 h, or 7 days of incubation with liquid bacterial cultures under static conditions, and the intensity of biofilm formation was determined by the crystal violet staining method. As a control for the growth of bacterial strains in liquid cultures, the optical density (OD_600_) of bacterial cultures in separate tubes was measured at the same time. All strains were able to form biofilms on urological catheters, but to a varying extent (Fig. 2). The statistical significance of biofilm formation was also different between incubation temperatures and catheter materials (Table 2). A more pronounced increase in biofilm mass over time was noted during incubation at 37 °C on latex catheters compared to silicone catheters, especially in the case of *E. hormaechei* strain 30345 (Fig. 2A), and *E. hormaechei* strain 29796, despite the decreased in its formation after 7 days (Fig. 2B), which confirms the results of other authors' research on biofilms produced by various species of bacteria and fungi (like *Candida albicans*) [67, 68]. This is presumably due to the smoother surface of silicone catheters, which makes it more difficult for bacteria to adhere [69, 70]. Interestingly, at room temperature, the biofilm produced by *E. hormaechei* strain 30345 adhered much more effectively to silicone catheters, whereas it was barely noticeable on latex catheter parts (Fig. 2A). The biofilm produced by *E. hormaechei* strain 29796 adhered similarly on latex catheters in both temperature conditions as well as on the silicone catheter at 37 °C, i.e., its mass increased over time for 4 days, while its production decreased after a week (the biofilm formed on silicone catheters at 24 °C was weakly marked and was not affected by the incubation time) (Fig. 2B). A similar tendency of *E. cloacae* biofilm formation on latex catheters was described by Misra et al. [18]. *E. hormaechei* strain 30528 was the least able to adhere in the form of biofilm to urological catheters, however, a tendency to increase the mass of biofilm on silicone catheters and to decrease it on latex catheters (except 24 °C) over time was noted (Fig. 2C). Misra et al. [18] described statistically significant more pronounced biofilm formation by *E. cloacae* on latex catheters at 37 °C when compared to room temperature. In addition, a more notable temperature-dependent biofilm adhesion on glass surfaces has been described [18]. In turn, *Cronobacter sakazakii* (formerly known as *Enterobacter sakazakii*) was able to form biofilm on enteral feeding tubes and stainless steel at 25 °C but not at 12 °C [71].

**Table 2:** Significance of differences in biofilm formation on urological catheters, depending on temperature and catheter material, generally for all times

Tukey’s multiple comparisons test	Significance	Summary	Adjusted *P* value
*Enterobacter hormaechei* strain 30345			
Silicone at 37 °C vs. latex at 37 °C	Yes	*	0.0195
Silicone at 24 °C vs. latex at 24 °C	Yes	****	< 0.0001
Silicone at 37 °C vs. silicone at 24 °C	Yes	**	0.0013
Latex at 37 °C vs. latex at 24 °C	Yes	****	< 0.0001
*Enterobacter hormaechei* strain 29796			
Silicone at 37 °C vs. latex at 37 °C	Yes	*	0.0176
Silicone at 24 °C vs. latex at 24 °C	No	ns	0.3394
Silicone at 37 °C vs. silicone at 24 °C	No	ns	0.6477
Latex at 37 °C vs. latex at 24 °C	No	ns	0.9982
*Enterobacter hormaechei* strain 30528			
Silicone at 37 °C vs. latex at 37 °C	Yes	**	0.0041
Silicone at 24 °C vs. latex at 24 °C	No	ns	0.0812
Silicone at 37 °C vs. silicone at 24 °C	No	ns	> 0.9999
Latex at 37 °C vs. latex at 24 °C	No	ns	0.6853

^*^p < 0.05; **p < 0.01; **** *p*< 0.0001; ns means not significant (*p* > 0.05)

All study strains grew similarly in liquid cultures, i.e., after 24 h of incubation, higher optical density (OD_600_) was noted at 37 °C, while over time it decreased, similar to observations described by Djeribi et al. [56], whereas OD_600_ increased in cultures incubated at room temperature during this time but decreased after a week (Fig. 2 A–C).

Only in some cases, the growth kinetics of liquid cultures correspond to the kinetics of the forming biofilm, i.e., *E. hormaechei* strain 29796 at 24 °C, on both catheters and *E. hormaechei* strain 30528 at 37 °C on latex catheter (Fig. 2B, C). However, the kinetics of the *E. hormaechei* strain 30528 biofilm formed on silicone at 24 °C is inversely proportional to the kinetics of the liquid culture (Fig. 2C).

It is worth noting that the limitation of this method is the deposition of bacterial biofilm on all parts of the cut catheters, i.e., apart from luminal and external surface [72], also on the transverse cutting surface, which does not occur under normal conditions. However, we noticed that the biofilm adherence at the cut surfaces was barely noticeable. Moreover, there are reports that biofilms on catheters may contain non-culturable bacteria that cannot be detected by standard methods [70].

Bacteria can grow in the form of biofilm on many different biotic and abiotic surfaces, and apart from the mentioned urinary catheters, they also pose a threat on dental materials [73, 74], nasogastric and orogastric enteral feeding tubes [75], prostheses [76], and other medical devices [77]. The mechanisms of bacterial adhesion to individual materials depending on surface properties and topography [78] are not fully understood, which makes research using different materials necessary. Recent single-cell force spectroscopy studies indicate that bacteria adhere to various standard surfaces, such as glass, and to various specially designed antifouling surfaces, using different adhesion forces and distinct mechanisms [79]. Moreover, although the general stages of biofilm formation remain unchanged, both the kinetics of biofilm formation and its architecture, as well as its individual components, may be specific to the bacterial species or even bacterial strain [80]. Moreover, the condition of individual biofilms is influenced to a varying extent by the presence of, for example, different ions, and different wettability and stiffness of the biofilm determine its adhesion and detachability [81].

### Screening of phage genomes for lysis-related phage enzymes

Numerous studies indicate the possibility of using bacteriophages as well as phage enzymes to inhibit the formation or degradation of bacterial biofilm matrices [35, 37, 82–85]. Initially, it was believed that biofilms acted as a barrier to bacteriophages due to the impermeability of the biofilm matrix. However, it has since been discovered that many bacteriophages can thrive within biofilms and actively replicate there. Bacteriophages employ a distinct mechanism for disrupting biofilms, different from that of antibiotics or biocides.

During the removal of biofilms by bacteriophages, two main mechanisms of action should be distinguished: the degradation of EPS (biofilm matrix) and the lysis of bacteria [85, 86]. The mechanism of biofilm degradation by phage-encoded enzymes (depolymerases and hydrolases) involves the specific breakdown of EPS, which form the structural matrix of biofilms [87, 88]. These enzymes include: glycoside hydrolases, SGNH/GDSL hydrolases, lytic transglycosylases, and NUDIX hydrolases. These enzymatic proteins target polysaccharides and other components of the biofilm matrix, reducing its integrity and facilitating the dispersion of bacterial cells. EPS degradation enhances the penetration of phages or other antimicrobial agents and is essential for dismantling the biofilm architecture, especially in the case of resistant bacterial strains [89]. The second mechanism is the lysis of susceptible bacterial cells, during which bacteriophages replicate within the host bacterial cells and cause their lysis, releasing progeny phages [90, 91]. Key lytic proteins involved in this process include: holins, antiholins, endolysins, and auxiliary lysis-inhibiting proteins, which degrade the bacterial cell wall (peptidoglycan layer), leading to cell rupture [92]. Bacterial lysis results in a rapid reduction of viable bacterial biomass and contributes to the collapse of the biofilm structure by destroying matrix-producing cells. Additionally, the self-propagating nature of this mechanism allows for deeper penetration into the biofilm as the infection progresses.

Both bacteriophages used in this study (i.e., Entb_43 and Entb_45) have been comprehensively characterized in previous studies [44]. In the Entb_43 phage genome (174,681 bp, circular dsDNA), 6 regions encoding proteins with lytic activity were identified, i.e.: SGNH/GDSL hydrolase family protein, glycoside hydrolase family protein, NUDIX hydrolase (as proteins associated with the degradation of biofilm structure), and lysis inhibition accessory protein, holin and antiholin (as proteins associated with bacterial cell lysis; Table 3). In turn, in the Entb_45 phage genome (172,771 bp circular dsDNA), 4 regions encoding proteins with lytic activity were identified, i.e.: lytic transglycosylase domain-containing protein, glycoside hydrolase family protein, NUDIX hydrolase and glycoside hydrolase family protein (proteins associated with the degradation of the biofilm structure; Table 3).

**Table 3:** Lysis-related enzymes detected in Entb_43 and Entb_45 phage genomes (based on RefSeq (GCF) genome assembly)

Protein ID	Locus tag	Gene type	CDS location in the genome			Gene product	Length [amino acids]	Structural peptide/amino acids sequence
			Orientation	Start	Stop			
Enterobacter phage Entb_43 (GenBank Acc. No. ON585039)								
UVD32386.1	ENTB43_255	protein-coding	Minus	7095	8630	SGNH/GDSL hydrolase family protein	511	MATDIWTTVHPIPWRNTSFLYLPWWIHNILIKAKEEGKDWREYADPQYALQIDTLKQAYVDYGEVNFIESKNGYQYTLSEYTVDGPDPQPPLGPMLQPVAVMSNPPTQKLAKGVGLSGTATIVSGGTGYVFGDIVDIPGGEGDTPGQIRVTAVGADGAITTAQIRKPGVYATAPTGEISATGGSGTGAKFTVTTNAGVASTIYAGVTISRTDAKITYWGSDIKDAVSGFRGNGTGNGTQCRVNFKTDSSKIDFKLAGNNSKYDLYVDGQRISATSVTTDSSGAVYIYSIDWNGVEQMREYSLVGVNTAFGGIYVDTGKTITAITRPAKMIWQLGDSYTFGTMATQASFNDFRFYCDKLGLVGLADGIGGSGWTSTSSTQPQARITAKLATLSFTPDIITLALGYNDAPAGRIDLLKTNFRESIALIKQYQPQAKIIVFGPATPLGMTDQIAAVRDALIELTTELDLEFVDVKGWVTAENANLYTSSDNVHPNDQGYFWRGSQFTNVLQDKV
UVD32490.1	ENTB43_053	protein-coding	Minus	59292	59567	antiholin	91	MTLRALAAILFAATLISPVSAEEANFEQYADGAMAVYSKFKEPSKEESERFFSFIKQKWAASSCTTQCTEEGVHAGKQYVSLTKVKLENEI
UVD32509.1	ENTB43_071	protein-coding	Minus	66114	66608	glycoside hydrolase family protein	164	MDIFGMLRIDEGYDSKIYKDTEGFWTIGIGHLLTRDPSLDVAKRELDKLVGRPCNGQITKAEAEAIFAKDVDKATRGILGNAVLKPVYDVLDGVRRAALINMVFQMGVAGVAGFPASMRLLKSKQWEAAAKELANSKWYRQTPNRAKRVIETFRVGTWKAYENL
UVD32510.1	ENTB43_072	protein-coding	Minus	66642	67121	NUDIX hydrolase	159	MSKKIKEVSAGIIFFTEDYELFMGRVTNSGLGAGPSRWDIPKGHIEEGETPIEAAIRECREETGFVDYDQSLLVDLGRHDYASNKDIHLFQYMHPVEHSQFRDCVCTAYHTDEDGNEFPEIDAFALIHPRMWNVVMGPSLFSVMQKLYPKVIQDALWEC
UVD32608.1	ENTB43_188	protein-coding	Minus	138297	138551	lysis inhibition accessory protein	84	MSMNKQLEHALHLQRNSWNAGHDNYGASIDVYAEALEVLKGFKHLNPVQADLRDVLALKDELKFAKPLCSAARKAVRHFVVTLK
UVD32654.1	ENTB43_233	protein-coding	Plus	167659	168315	holin	218	MTQRTPLPGISDILFGVLDRLFKDNATGRVLASRVVALVVVFILSLTWYRLDSIMQVWKESRYETYTKVLQQDKEAKFEASALEQLQIAHVSSNADFSAIYSFRPRNLNYFVDLIAYEGRLPSAVNEKNLGGFPVDKTSNEYSAHLRGAYFSSENEFVFLPTKKKDGELKYMYSCPYFNLDNVYAGTVSMYWYSKPLLNEDRLAAICGQAARTLGRAK
Enterobacter phage Entb_45 (GenBank Acc. No. ON630910)								
UTY64352.1	ENTB45_172	protein-coding	Minus	59305	59856	lytic transglycosylase domain-containing protein	183	MKKVLATLLLTVSMSAHSVEPTFSNEQLDNLQYAYAFGEQFQKSGKFKEPSKRYDNNGLGYIMAGLAWQESSAGLNTGEEKHKHHAYGMFQNYLPTLRNRMAQVGWKMSDKEIIKMIKERKNSASWAYIELSYWLERHNGDMRKAIASYNAGNNVKAGNKYASQVLAKANYLKSKRMLHQTVD
UTY64363.1	ENTB45_183	protein-coding	Minus	64382	65333	glycoside hydrolase family protein	162	MDIFGMLRIDEGCKLELYKDTEGFWTIGIGQLITKNPSYNVARDELDRLMGRVCNGRITQQEAENLFNSSVEKARKGILGNATLKPVYDVLDEVRRCALINMVFQMGVAGTAGFPKGMRLLKAKQWDKAAIELADSRWYKQTPNRAKRVILTFKTGTWAAYR
UTY64364.1	ENTB45_184	protein-coding	Minus	64845	66019	NUDIX hydrolase	136	MGRVTNSSLRSGMPSRWDIPKGHIEEGETALEAAVRECREETGFIDYNPALLVDLGRQKYASNKDLHVFVYPIPVRHEQFKDCVCTAYHEDRETGERFPEIDAFALIKPSQWNYVMGPSLFNVMQRLFPKESKSVG
UTY64401.1	ENTB45_229	protein-coding	Plus	82267	84012	glycoside hydrolase family protein	581	MINISDSVSWFVGVVEDRMDPLEQGRVRVRVWGLHPYEKTQGPVKGIKTEDLPWMSVLQPTSSASVSGVQQAITGMVPGTHVYGHFLDKWKLNGLVLGTYSSNSMVKANPNEGFADPTGQYPLYLGNDTNPLNRGGIPGDDSTVNIIQDANLDVGINPDGKPLSEIPEDNNPNYTIQAMVQHDEGLRLKVYRDLNGRGWTVGFGHYISDNPNLTNDQINSELSKQVGREVTGNPGSISMDEASKLFKEDLEKVQRDMRNNAAVSPVYMKVNRSRQMALENMAFQLGIGGLAKFTNMLEAMFIGDWTTAYKEARNSLWFNQTKGRASRVSMIILTGNMESYGIPVNPNQNPNGRMVSFATLAAEPVASDPADPWVPEDTRILFKEPPSSYKGQYPYVQTMATEGGHIQEFDNTPGQERYRLIHPTGSYDETAPDGRRTIKSVADAYYMTQADCSTMIGGDNKVNVGGNEVKYNMADVRRQTDGNETIFIRGNDTKTVEGDGTLHVKGNIKIIVEGDADIEVQGNAKSHVVGNHEYTVDGNLTWKIAGTVNIDVGGSWTEKFASMNSVASGQYTIDGSRIDIG

Holins are small hydrophobic proteins that accumulate in the inner cell membrane and, upon oligomerization, form pores [93]. This pore formation triggers the activation of peptidoglycan hydrolase (endolysin), which accumulates in the periplasm or cytoplasm and breaks down the bacterial cell wall at the end of the lytic cycle [93, 94]. The specificity of these enzymes, like the phages that produce them, is generally high [83, 95]. When combined with the fagolin protein, these enzymes lead to the lysis of the bacterial cell from within [83]. Phage depolymerases, which vary in activity, are strain-specific enzymes and present a challenge for eradicating mixed biofilms. Another class of phage enzymes is DNases, which degrade bacterial DNA when released into the environment [36]. A further group consists of phage tail enzymes, although their activity is limited since they are often masked until the phage tail undergoes reconfiguration during infection [83].

The main advantage of using phage enzymes is the lack of risk for horizontal gene transfer (HGT) between bacterial populations as well as the likely absence of the risk of inducing bacterial resistance to these enzymes [96]. However, a key disadvantage is that these enzymes must be applied repeatedly to the biofilm, whereas phages naturally amplify their concentration through the infection of bacterial host cells [97]. Distinguishing between EPS degradation by phages and bacterial lysis is crucial for designing effective anti-biofilm strategies. EPS breakdown alone may be insufficient, as bacterial cells can remain viable, while lysis without EPS penetration tends to be inefficient. EPS-degrading enzymes expand the applicability of phages in resistant or mixed-species biofilms, whereas lytic activity ensures the reduction of viable pathogens. The combination of both mechanisms, through synergistic action, significantly contributes to the destruction of the biofilm structure [91].

### Phages disrupt biofilm formation

**Fig. 3:**
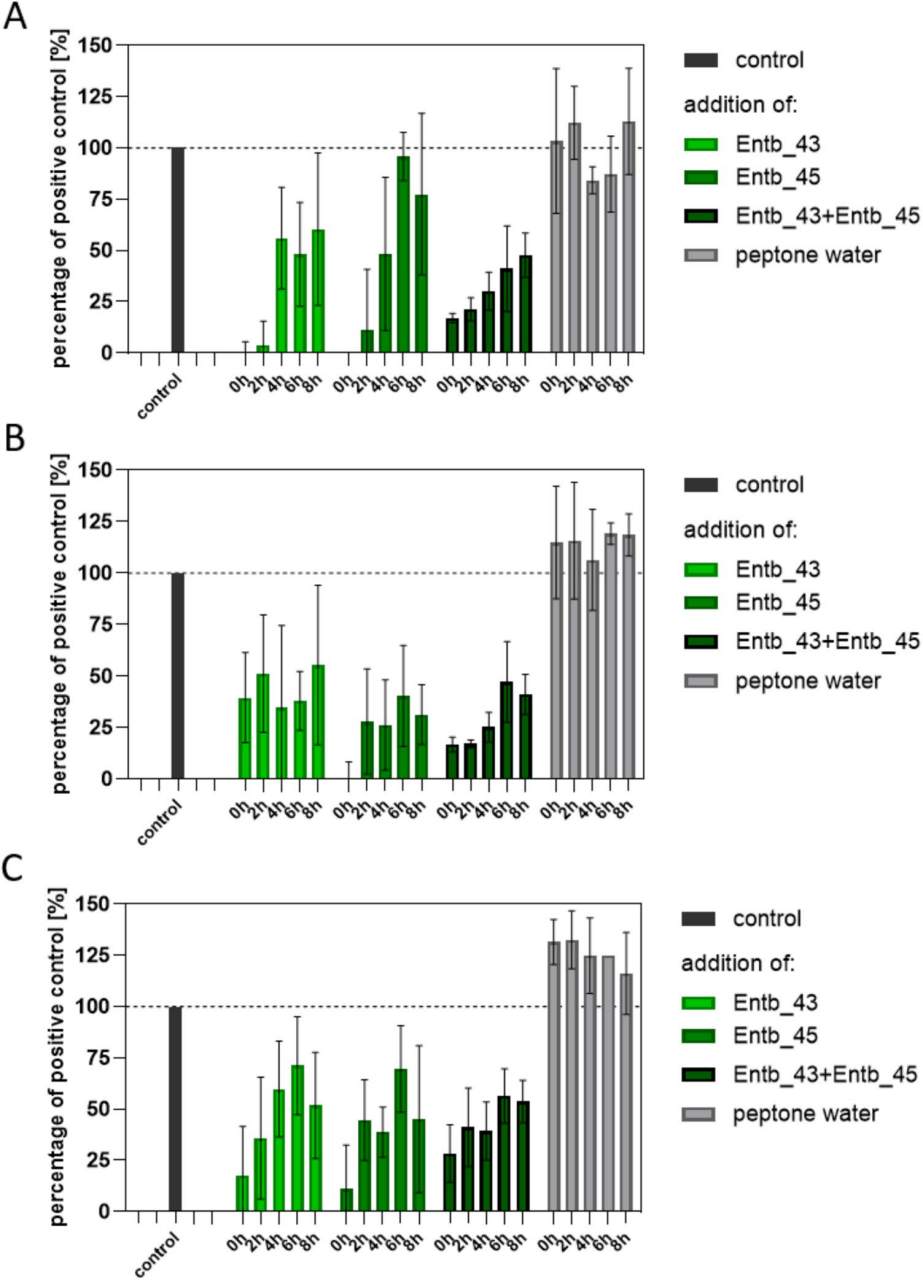
Disruption of biofilm formation by application of phage lysates. Biofilms were formed by **A** *E. hormaechei* strain 30345; **B** *E. hormaechei* strain 29796; or **C** *E. hormaechei* strain 30528. Bacteriophages, phage cocktail, or peptone water were applied at different time points during biofilm formation (0 h, 2 h, 4 h, 6 h, or 8 h; *n* = 14 for each group). Results referred to bacteria that grew undisturbed for 24 h and were expressed as 100%

We decided to assess whether bacteriophage application during biofilm formation may disrupt this process. For this purpose, phage lysates or their cocktail were added to bacteria suspensions at different times (0 h, 2 h, 4 h, 6 h, or 8 h) during incubation. Simultaneously, peptone water without phages was applicate to control wells. The positive control considered as 100% biofilm formation was formed by bacterial suspensions intact cultured for 24 h (Fig. 3). In each case, application of Entb_43 phage, Entb_45 phage, or phage cocktail disrupted biofilm formation, especially when used in early stages of this process. Biofilm formation by *E. hormaechei* strain 30345 was most inhibited by Entb_43 phage, for which it is a host, and by a mixture of both phages (Fig. 3A). Similarly, Entb_45 phage inhibited biofilm especially produced by its host, *E. hormaechei* strain 29796 (Fig. 3B). Both phages disrupted biofilm formation by *E. hormaechei* strain 30528 to a similar extent (Fig. 3C). The inhibition of biofilm formation by application of all phage combinations for all strains was statistically significant (Table 4). Similar observations were described for the *E. cloacae*-specific En5822 phage, where the phage (with a titer of 10^3^ PFU/mL) was applied at the beginning of biofilm formation by its bacterial host. At different time points, from 4 h to 72 h, a clear reduction in biofilm formation was observed, up to 95%, while the biofilm mass increased over time in the untreated sample [48]. Interestingly, the addition of peptone water at specific times instead of phages, had a positive effect on the condition of bacteria and biofilm mass increase in most cases (Fig. 3A–C).

**Table 4:** Significance of differences in disruption of biofilm formation by application of phage lysates, generally for all times

Dunnett’s multiple comparisons test	Significance	Summary	Adjusted *P* value
*Enterobacter hormaechei* strain 30345			
Control vs. Entb_43	Yes	**	0.0099
Control vs. Entb_45	Yes	**	0.0016
Control vs. Entb_43+Entb_45	Yes	**	0.0011
Control vs. peptone water	No	ns	0.9999
*Enterobacter hormaechei* strain 29796			
Control vs. Entb_43	Yes	****	< 0.0001
Control vs. Entb_45	Yes	****	< 0.0001
Control vs. Entb_43+Entb_45	Yes	****	< 0.0001
Control vs. peptone water	No	ns	0.423
*Enterobacter hormaechei* strain 30528			
Control vs. Entb_43	Yes	****	< 0.0001
Control vs. Entb_45	Yes	****	< 0.0001
Control vs. Entb_43+Entb_45	Yes	****	< 0.0001
Control vs. peptone water	Yes	*	0.0139

* *p* < 0.05; ** *p* < 0.01; **** *p* < 0.0001; ns means not significant (*p* > 0.05)

### Bacteriophages and nanoparticles destroy biofilm formed by *E. hormaechei*

Silver nanoparticles have been described as potent anti-biofilm agents, due to different possible mechanisms of action, e.g., disruption in bacterial plasma membrane permeability, inactivation of bacterial enzymes, induction of reactive oxygen intermediates (ROIs) production [98]. Copper and copper-derived materials are also known to have antibacterial properties [99], but some studies indicate that this direct effect of copper nanoparticles is negligible [100]. We decided to assess the anti-biofilm effect of both phages, their cocktail, as well as commercially available silver and copper nanoparticles alone and in combination with bacteriophages. Moreover, we tested the ability of bacteriophages to destroy a three-day biofilm formed by *E. hormaechei* on urological catheters made of different materials (silicone or latex). After this incubation time, an irreversible stage of biofilm formation should be reached [101].

**Fig. 4:**
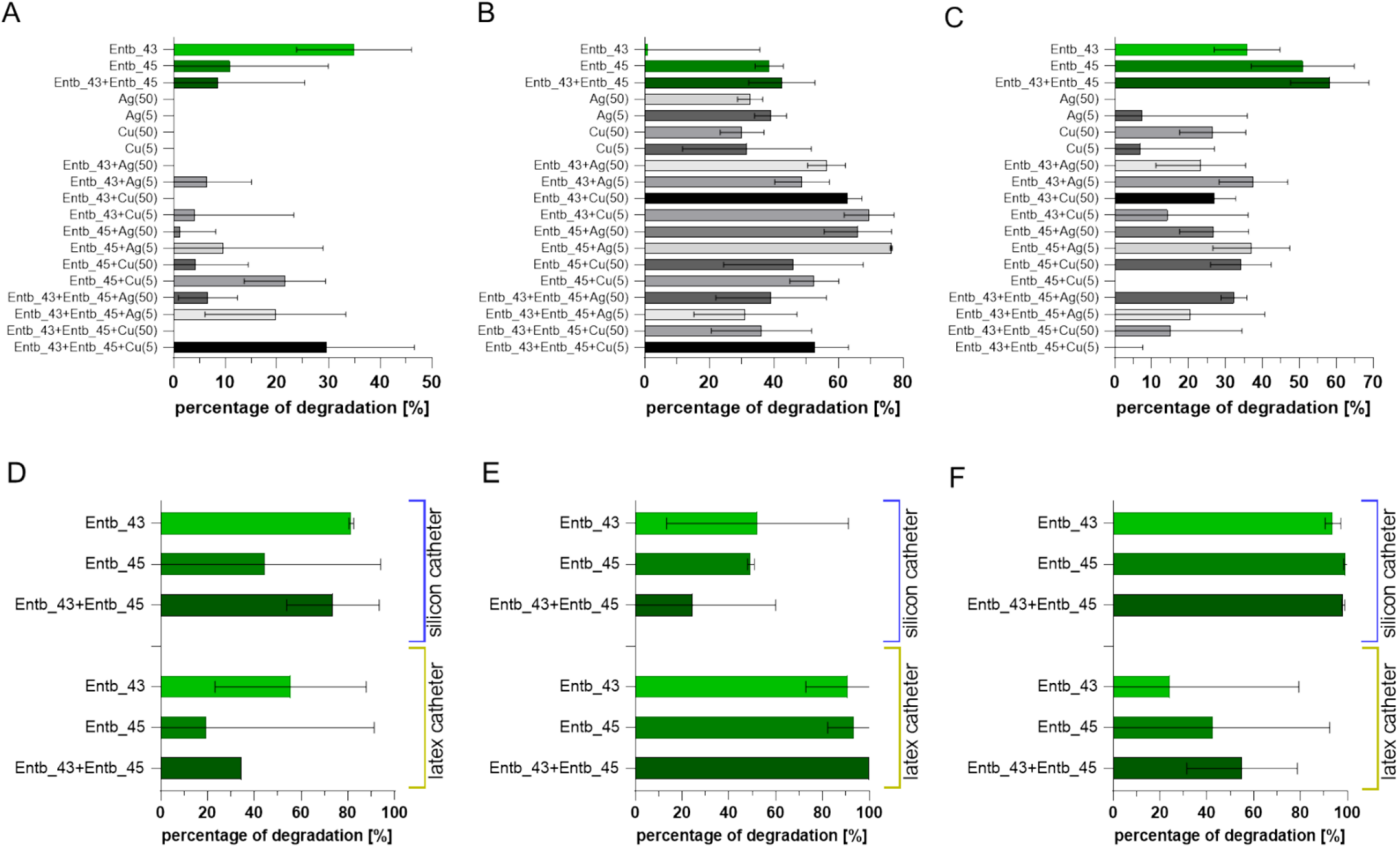
The ability of phages to destroy existing biofilm formed by different strains of *E. hormaechei*. The upper panel shows the anti-biofilm activity of *Enterobacter*-specific phages or/and silver or copper nanoparticles as a percentage of biofilm degradation. The lower panel shows the degradation of biofilm formed on silicon or latex urological catheters. Anti-biofilm factors were tested on biofilms produced by *E. hormaechei* strain 30345 (**A** and **D**), *E. hormaechei* strain 29796 (**B** and **E**), or *E. hormaechei* strain 30528 (**C** and **F**). In the standard experiment (upper panel), tested factors include Entb_43 phage, Entb_45 phage, cocktail of both phages, silver nanoparticles in two concentrations (5 or 50 mg/kg), copper nanoparticles in two concentrations (5 or 50 mg/kg), mixture of each phage with each nanoparticle and phage cocktail with nanoparticles (*n* = 4 in each group). In the catheter studies (lower panel), both phages were used separately and as a phage cocktail (*n* = 6 in each group). Error bars represent the standard deviation (± SD) of the mean calculated values

Destruction of biofilm by phages and/or silver or copper nanoparticles was strain-dependent. *Enterobacter* strains differ in particular in their sensitivity to nanoparticles (Fig. 4A–C). Both metal nanoparticle preparations also differ in their influence on the anti-biofilm effect of individual bacteriophages (Supplementary Table S2). The highest *E. hormaechei* strain 30345 biofilm-degrading activity of 35% was observed for Entb_43 phage, of which this bacterium is a host (Fig. 4A). The application of the phage cocktail reduced the biofilm only by about 12%, which may be related to the antagonistic action of both phages, similar to the results described by Namonyo et al. [102], where none of the phage cocktails used was effective against the *P. aeruginosa* biofilm. Similarly, studies of our group on biofilm formed by *A. baumannii* showed that single phages were more effective than a phage cocktail [45]. However, the phage cocktail combined with antibiotics was much more effective in disrupting *A. baumannii* biofilm than antibiotics alone [103]. In the present study, there was no effect of nanoparticles alone on the destruction of *E. hormaechei* strain 30345 biofilms, as their presence enhanced its formation (data not presented in the graph because it referred to the positive control, which consisted of bacteria not influenced by any factors). Moreover, the presence of nanoparticles disrupted the activity of Entb_43 phage against biofilm formed by *E. hormaechei* strain 30345 (Fig. 4A), although we had previously demonstrated a statistically significant decrease in the titer of this phage following 24 h of incubation with nanoparticles at room temperature, but it was only max. 1 order of magnitude [44]. It is also worth mentioning that a statistically significant, but less pronounced, decrease in Entb_43 phage titer was observed after 30 min of incubation with nanoparticles [44], therefore it can be suspected that this bacteriophage was degraded by them before it interacted with the biofilm. However, the addition of each of the nanoparticles at a lower concentration used (5 mg/kg) to the Entb_45 phage or to the cocktail of both phages enhanced their anti-biofilm activity (Fig. 4A).

In turn, biofilm formed by *E. hormaechei* strain 29796 was degraded by Entb_45 phage in 38.6%, for which it is a host, and despite the very weak anti-biofilm activity of Entb_43 phage (about 1%), the cocktail of both phages had a more effective degrading effect (42.5%) (Fig. 4B). Moreover, the biofilm produced by this strain is more sensitive to the anti-biofilm activity of silver and copper nanoparticles, which supported the anti-biofilm activity of phages (Fig. 4B). Particularly, the addition of silver or copper nanoparticles to Entb_43 phage showed statistically significant differences in anti-biofilm activity compared to phage-only preparations (Supplementary Table S2).

Biofilm produced by *E. hormaechei* strain 30528 was the most effectively degraded by either Entb_45 phage or a cocktail of both phages, i.e., 51% or 58% reduction in biofilm mass, respectively (Fig. 4C). In almost all cases (except the application of a phage cocktail with silver nanoparticles), a more effective anti-biofilm activity was observed when a higher dose of copper nanoparticles (50 mg/kg) or a lower dose of silver nanoparticles (5 mg/kg) was applied (Fig. 4C). However, except for one case, the addition of nanoparticles to bacteriophages did not result in statistically significant differences compared to preparations containing only phages (Supplementary Table S2). It is worth mentioning that the *E. hormaechei* strain 30528 is resistant to the lysis by Entb_43 phage in the spot test ([44]; the tests were also repeated in the current study), which may indicate the EPS-degrading activity of lytic enzymes produced by this phage (Table 3). However, it can be suspected that the highest noted level of degradation of the biofilm produced by this strain (almost 60%) by the phage cocktail could result from the complementation of the direct lytic of bacterial cells by Entb_45 phage and the destruction of EPS by Entb_43 phage enzymes (Fig. 4C and Table 3).

Jamal et al. [104] described the significant reduction of biofilm formed by multidrug resistant *E. cloacae* after just 4 h of incubation with bacteriophage. In a study conducted by Nair et al. [48], the kinetic of existing biofilm reduction following phage application was visualized, and after 72 h it reached 76%.

**Fig. 5:**
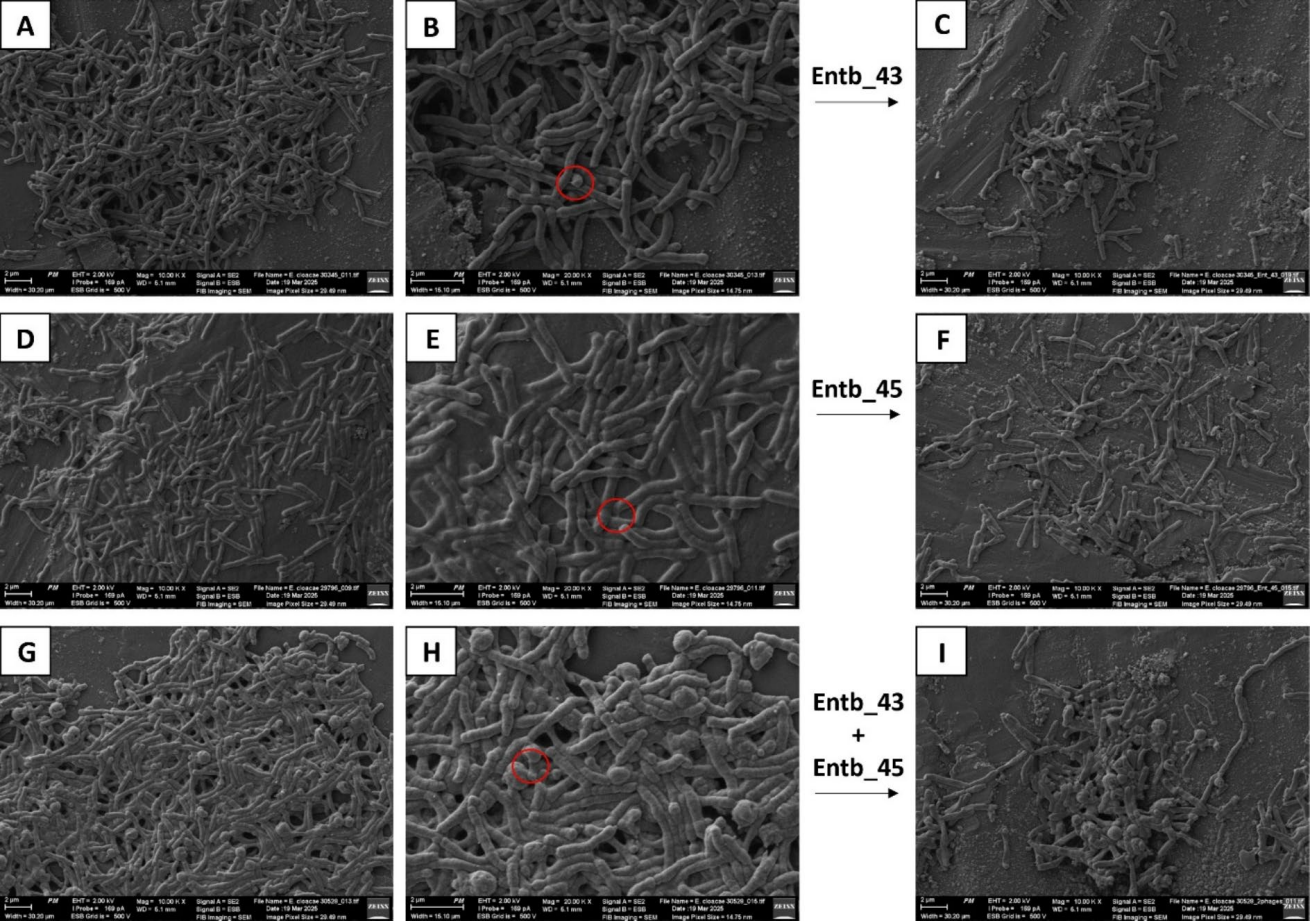
SEM images showing bacterial biofilm formed by clinical isolates of *E. hormaechei* before and after exposure to bacteriophages. The images show biofilm plaques produced by *E. hormaechei* strain 30345 (without exposure to phages: **A, B**; after exposure to Entb_43 phage: **C**), *E. hormaechei* strain 29796 (without exposure to phages: **D, E**; after exposure to Entb_45 phage: **F**), *E. hormaechei* strain 30528 (without exposure to phages: **G, H**; after exposure to cocktail of both phages: **I**). The **A** and** C**; **D** and** F**; **G** and** I** images represent a magnification of 10,000×, while the **B, E, H** images represent a magnification of 20,000×. The bars at the bottom of the images represent 2 µm. The red circle marks the characteristic nanotube-like structures between the cells forming the biofilm

Furthermore, biofilms formed by the three *E. hormaechei* isolates on polystyrene 96-well plates were visualized using SEM (Fig. 5). *E. hormaechei* strains 30345 and 30528 developed well-defined lamellar biofilm structures (Fig. 5A, G). In contrast, although the biofilm produced by *E. hormaechei* strain 29796 appeared less compact, its most prominent characteristic was the extensive EPS matrix enveloping the bacterial cells (Fig. 5D). At a higher magnification (20,000×), characteristic nanotube-like structures connecting the biofilm-forming cells can be identified (Fig. 5B, E, H); these structures are involved in nutrient exchange during the early stages of biofilm development and contribute to maintaining close cell-to-cell contact [18]. The structures of biofilms were also visualized after 24 h of incubation with selected bacteriophage preparations: biofilm formed by *E. hormaechei* strain 30345 with Entb_43 phage (Fig. 5C), biofilm formed by *E. hormaechei* strain 29796 with Entb_45 phage (Fig. 5F), as well as biofilm formed by *E. hormaechei* strain 30528 with cocktail of both phages (Fig. 5I). In all cases, a disruption of the biofilm architecture was noted, and the remaining bacterial cells exhibited clear morphological alterations (Fig. 5C, F, I).

In the case of the anti-biofilm effect of phages on the biofilm formed on catheters by *E. hormaechei* strain 30345, similar trends were observed for both types of catheters, i.e., the greatest effect was shown by Entb_43 phage (similarly as indicated above), then the phage cocktail and the weakest effect was noted for Entb_45 phage (Fig. 4D). Surprisingly, both bacteriophages and their combination demonstrated almost complete degradation of the existing biofilm formed by *E. hormaechei* strain 29796 on latex catheters (Fig. 4E) and by *E. hormaechei* strain 30528 on silicone catheters (Fig. 4F). However, such marked results in comparison with the experiment described above may result from the fact that biofilms formed on catheters had lower absorbance values, resulting, among others, from the relatively high adhesion of crystal violet to latex, which caused high values to be subtracted from the tested samples. Moreover, the lack of the gene encoding the FimH adhesin in all tested strains (Fig. 1C) may result in relatively weak adhesion of the biofilm to catheters (Fig. 2), as mentioned above. Interestingly, the same trend of phage activity as in the above experiment was noted for the biofilm formed by *E. hormaechei* strain 30528 on a latex catheter (Fig. 4F).

Various strategies are used to prevent biofilm formation and include, for example, coating surfaces with nanostructured graphene [101], coating with antibacterial agents like antibiotics, or various antiseptics [105], as well as modifying the material surface to prevent bacterial adhesion, which is the first step in biofilm formation [106]. Pretreatment of urinary catheters with phages has been found to reduce the level of single- and double-species biofilms [107]. Similarly, various methods are being used and investigated to degrade biofilm and treat biofilm-associated infections, and one of them is bacteriophage-based methods, with particular focus on biofilm-forming Gram-negative bacteria [35, 108, 109]. Phage therapy is considered safe [110, 111], therefore methods using bacteriophages to prevent the formation and degradation of existing biofilm, also in combination with other antimicrobials, seem worthy of further investigation.

## Conclusions

Most of the clinical multidrug-resistant *Enterobacter* strains presented in this study were able to create biofilm. The *fimA*, *csgA*, *csgD*, and *sdiA* genes, which are associated with biofilm formation, were present in all three strains tested. These *E. hormaechei* strains were capable of producing biofilm on urological catheters, and this process depended on the type of material from which the catheter was made, as well as on the time and temperature of incubation. Positive results were obtained both at human body temperature and at room temperature, where catheters are typically stored, highlighting the risk of catheter-related infections. Incubation with *Enterobacter*-specific bacteriophages enabled, in some cases, almost complete eradication of biofilms attached to catheters. Moreover, commercially available silver or copper nanoparticles affected the anti-biofilm properties of phages to a varying extent in standard tests. Interestingly, both phage lysates and their cocktail were able to degrade biofilm produced by strain insensitive to a given phage, which may indicate the action of lysis-related enzymes detected in phage genomes. Furthermore, inhibition of biofilm formation by phage application during this process has been proven. These results highlight the threat of biofilm-related infections, but also indicate the multifaceted anti-biofilm activity of bacteriophages, which could be useful in clinical practice.

## Supplementary Information

Below is the link to the electronic supplementary material.

Supplementary file1 (DOCX 25 kb)

## Data Availability

The *16S* rDNA gene region sequences have been deposited in the GenBank database under accession numbers PQ785782, PQ785787 and PQ785788 for *E. hormaechei* strain 29796, *E. hormaechei* strain 30345 and *E. hormaechei* strain 30528, respectively. The complete genomes of the Enterobacter phage Ent_43 and Enterobacter phage Ent_45 have been sequenced and deposited in the GenBank database under the accession numbers ON585039 and ON630910, respectively. Other data will be made available on request.

## Abbreviations

CFU: Colony-forming unit
dsDNA: Double-stranded deoxyribonucleic acid
ECC: *Enterobacter cloacae* complex
EPS: Extracellular polymeric substance
HAI: Healthcare-associated infection
HGT: Horizontal gene transfer
MALDI-TOF MS: Matrix-assisted laser desorption/ionization time-of-flight mass spectrometry
MDR: Multidrug-resistant
NCBI: National Center for Biotechnology Information
PBS: Phosphate-buffered saline
PFU: Plaque-forming unit
ROI: Reactive oxygen intermediate
RTD: Routine test dilution
SEM: Scanning Electron Microscope
UTI: Urinary tract infection
WHO: World Health Organization
