# Supplementary material for: Fighting biofilm: bacteriophages eliminate biofilm formed by multidrug-resistant Enterobacter hormaechei on urological catheters
Source: Med Microbiol Immunol. 2025 Jul 3;214(1):33. doi: 10.1007/s00430-025-00844-0 (PMC12226686; doi:10.1007/s00430-025-00844-0)
Supplement: Supplementary file 1 — Supplementary file1 (DOCX 25 kb) [file 430_2025_844_MOESM1_ESM.docx]

Supplementary materials for

**Fighting Biofilm: Bacteriophages Eliminate Biofilm Formed by Multidrug-Resistant
*Enterobacter hormaechei* on Urological Catheters**

Martyna Cieślik^†^, Michał Wójcicki^†^, Paweł Migdał, Ilona Grygiel, Olaf Bajrak, Filip Orwat, Andrzej Górski,
Ewa Jończyk-Matysiak^*^

**Table S1** The ability to produce biofilms by twenty *Enterobacter* spp. strains after 24 h or 48 h of incubation at 37 °C. Assessment of biofilm formation were performed using method described by Stepanović et al. [49]

| **No.** | **Bacterial Strain**  **(identification according**  **to MALDI-TOF MS)** | **Incubation time** | |
| --- | --- | --- | --- |
|  |  | **24 h** | **48 h** |
| 1. | *E. cloacae* strain 30345* | moderate | moderate |
| 2. | *E. cloacae* strain 29796* | weak | weak |
| 3. | *E. cloacae* strain 3344 | weak | weak |
| 4. | *E. cloacae* strain 3345 | weak | weak |
| 5. | *E. cloacae* strain 30697 | moderate | moderate |
| 6. | *E. cloacae* strain 30528* | weak | weak |
| 7. | *E. cloacae* strain 30103 | - | - |
| 8. | *E. cloacae* strain 29916 | weak | weak |
| 9. | *E. cloacae* strain 29779 | weak | moderate |
| 10. | *E. cloacae* strain 29731 | - | weak |
| 11. | *E. cloacae* strain 30642 | weak | moderate |
| 12. | *E. cloacae* strain 30256 | - | - |
| 13. | *E. cloacae* strain 3354 | weak | - |
| 14. | *E. cloacae* strain 30612 | weak | weak |
| 15. | *E. cloacae* strain 30300 | moderate | weak |
| 16. | *E. hormaechei* strain 30165 | moderate | weak |
| 17. | *E. hormaechei* strain 30426 | weak | - |
| 18. | *E. hormaechei* strain 30550 | weak | weak |
| 19. | *E. hormaechei* strain 29753 | moderate | moderate |
| 20. | *E. kobei* strain 30367 | strong | strong |

***** based on *16S* rDNA gene region sequencing, this strain was reclassified as *Enterobacter hormaechei*

**Table S2** Statistical differences in the anti-biofilm activity of phage preparations with/without the addition of silver or copper nanoparticles. One-way ANOVA and Tukey’s multiple comparison test were performed. ** *p* < 0.01; *** *p* < 0.001; **** *p* < 0.0001; ns means not significant (*p* > 0.05)

| ***Enterobacter hormaechei*** **strain 30345** | | | |
| --- | --- | --- | --- |
| **Tukey’s multiple comparisons test** | **Significance** | **Summary** | **Adjusted P value** |
|  | | | |
| Entb_43 vs. Entb_43+Ag(50) | Yes | ** | 0.0059 |
| Entb_43 vs. Entb_43+Ag(5) | No | ns | 0.1180 |
| Entb_43 vs. Entb_43+Cu(50) | Yes | ** | 0.0059 |
| Entb_43 vs. Entb_43+Cu(5) | No | ns | 0.0593 |
|  | | | |
| Entb_45 vs. Entb_45+Ag(50) | No | ns | 0.9996 |
| Entb_45 vs. Entb_45+Ag(5) | No | ns | > 0.9999 |
| Entb_45 vs. Entb_45+Cu(50) | No | ns | > 0.9999 |
| Entb_45 vs. Entb_45+Cu(5) | No | ns | 0.9994 |
|  | | | |
| Entb_43+Entb_45 vs. Entb_43+Entb_45+Ag(50) | No | ns | > 0.9999 |
| Entb_43+Entb_45 vs. Entb_43+Entb_45+Ag(5) | No | ns | 0.9947 |
| Entb_43+Entb_45 vs. Entb_43+Entb_45+Cu(50) | No | ns | > 0.9999 |
| Entb_43+Entb_45 vs. Entb_43+Entb_45+Cu(5) | No | ns | 0.4416 |
|  | | | |
| ***Enterobacter hormaechei* strain 29796** | | | |
| **Tukey’s multiple comparisons test** | **Significance** | **Summary** | **Adjusted P value** |
|  | | | |
| Entb_43 vs. Entb_43+Ag(50) | Yes | ** | 0.0021 |
| Entb_43 vs. Entb_43+Ag(5) | Yes | ** | 0.0077 |
| Entb_43 vs. Entb_43+Cu(50) | Yes | *** | 0.0003 |
| Entb_43 vs. Entb_43+Cu(5) | Yes | **** | < 0.0001 |
|  | | | |
| Entb_45 vs. Entb_45+Ag(50) | No | ns | 0.4409 |
| Entb_45 vs. Entb_45+Ag(5) | No | ns | 0.0552 |
| Entb_45 vs. Entb_45+Cu(50) | No | ns | > 0.9999 |
| Entb_45 vs. Entb_45+Cu(5) | No | ns | 0.9922 |
|  | | | |
| Entb_43+Entb_45 vs. Entb_43+Entb_45+Ag(50) | No | ns | > 0.9999 |
| Entb_43+Entb_45 vs. Entb_43+Entb_45+Ag(5) | No | ns | 0.9997 |
| Entb_43+Entb_45 vs. Entb_43+Entb_45+Cu(50) | No | ns | > 0.9999 |
| Entb_43+Entb_45 vs. Entb_43+Entb_45+Cu(5) | No | ns | 0.9998 |
|  | | | |
| ***Enterobacter hormaechei* strain 30528** | | | |
| **Tukey’s multiple comparisons test** | **Significance** | **Summary** | **Adjusted P value** |
|  | | | |
| Entb_43 vs. Entb_43+Ag(50) | No | ns | 0.9993 |
| Entb_43 vs. Entb_43+Ag(5) | No | ns | > 0.9999 |
| Entb_43 vs. Entb_43+Cu(50) | No | ns | > 0.9999 |
| Entb_43 vs. Entb_43+Cu(5) | No | ns | 0.8582 |
|  | | | |
| Entb_45 vs. Entb_45+Ag(50) | No | ns | 0.7277 |
| Entb_45 vs. Entb_45+Ag(5) | No | ns | 0.9991 |
| Entb_45 vs. Entb_45+Cu(50) | No | ns | 0.9918 |
| Entb_45 vs. Entb_45+Cu(5) | No | ns | 0.2465 |
|  | | | |
| Entb_43+Entb_45 vs. Entb_43+Entb_45+Ag(50) | No | ns | 0.7278 |
| Entb_43+Entb_45 vs. Entb_43+Entb_45+Ag(5) | No | ns | 0.1415 |
| Entb_43+Entb_45 vs. Entb_43+Entb_45+Cu(50) | No | ns | 0.0874 |
| Entb_43+Entb_45 vs. Entb_43+Entb_45+Cu(5) | Yes | ** | 0.0029 |
